# Supplementary material for: Machine-learning media bias
Source: PLoS One. 2022 Aug 10;17(8):e0271947. doi: 10.1371/journal.pone.0271947 (PMC9365193; doi:10.1371/journal.pone.0271947)
Supplement: S1 Appendix — (PDF) [file pone.0271947.s001.pdf]

S1 Appendix: Supporting Information

In this section, we include supplementary technical details in the form of additional generalized eigenvalue plots and topic relevance weights. Underlying data can be found at the following link: https://space.mit.edu/home/tegmark/phrasebias.html

Figure S 1. Affirmative action bias

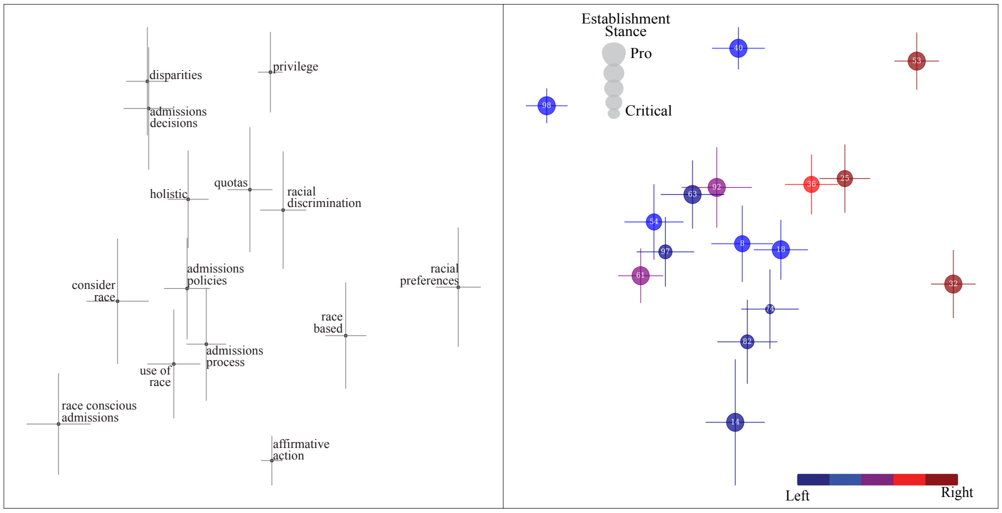

Figure S 2. Palestine bias

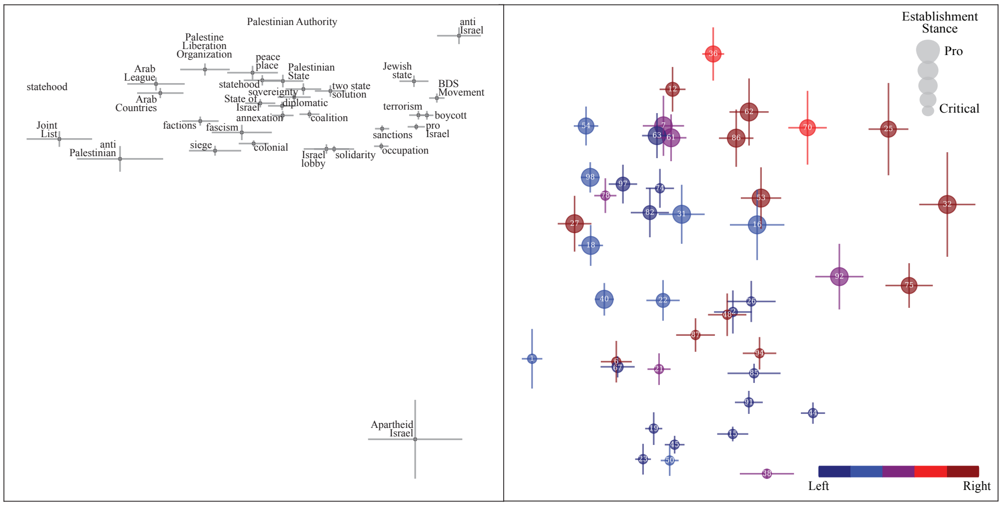

Figure S 3. Public finance bias

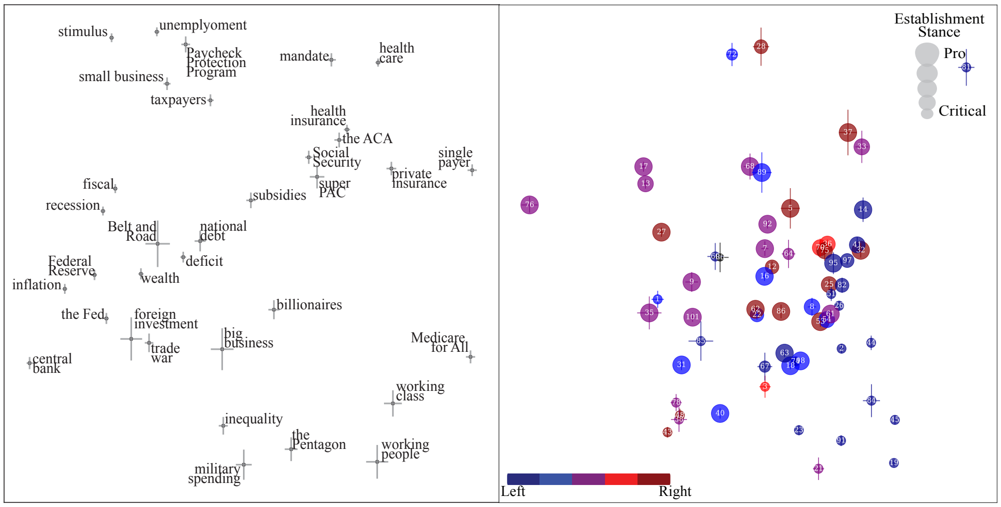

Figure S 4. Human rights bias

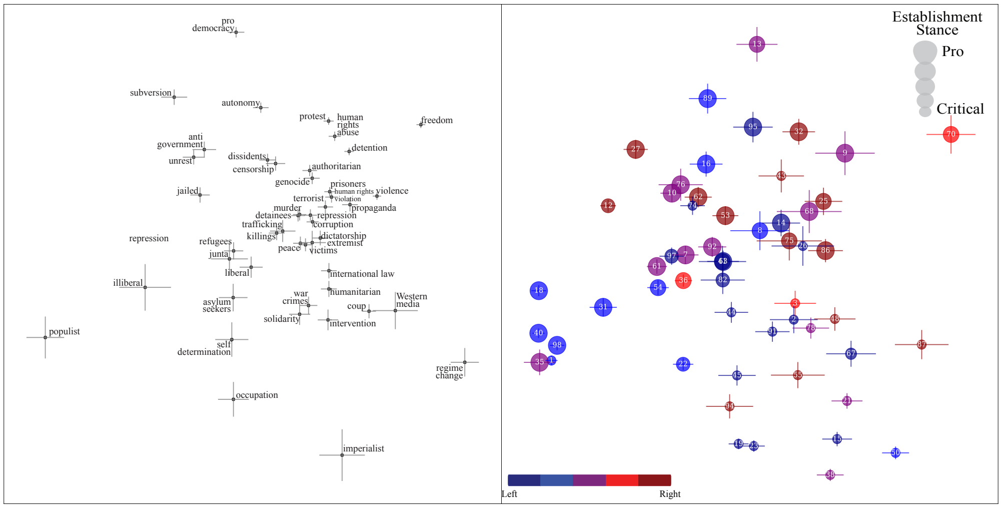

Figure S 5. Israel bias

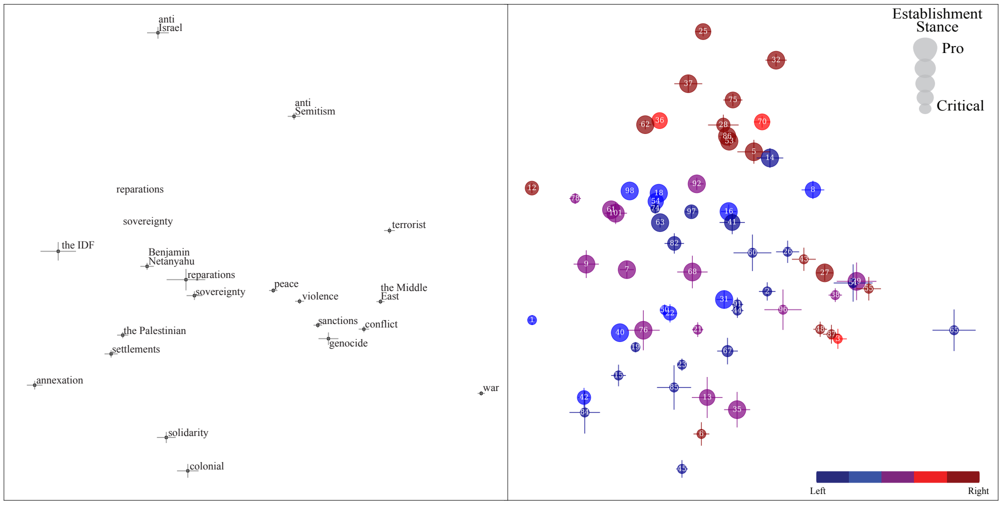

Figure S 6. Private finance bias

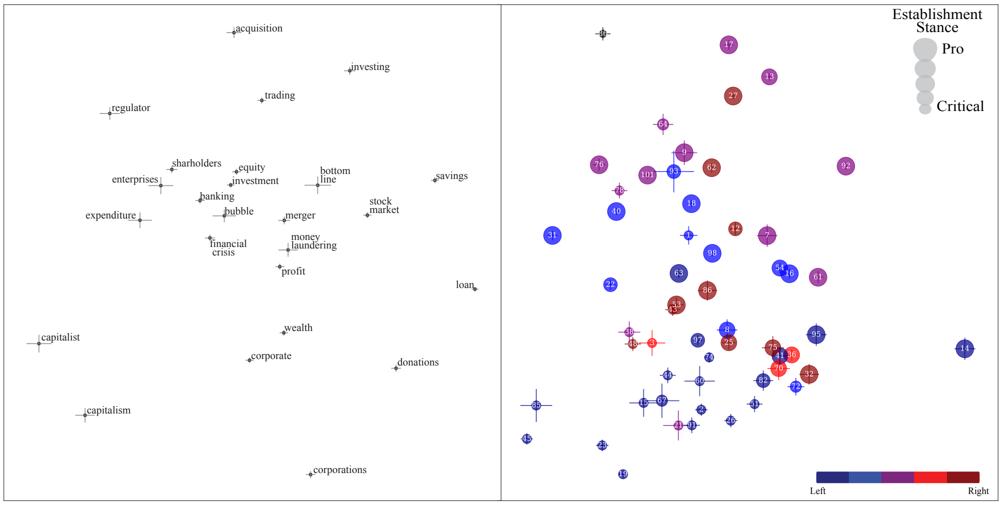

Figure S 7. Native Americans bias

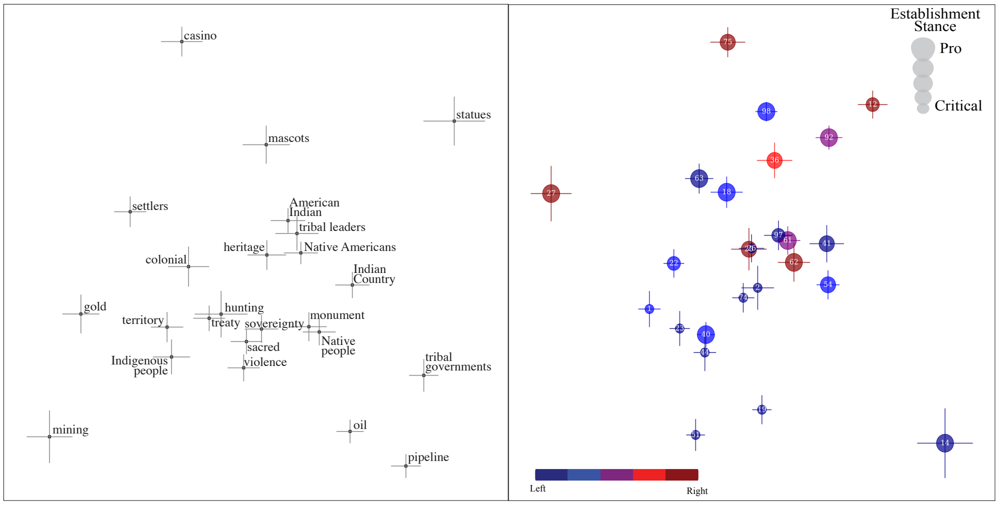

Figure S 8. Oil bias

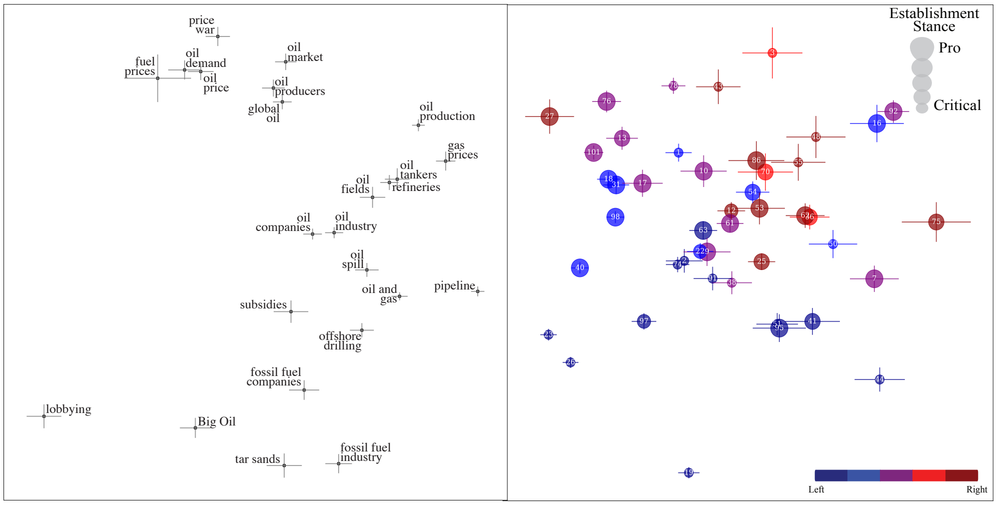

Figure S 9. Prisons bias

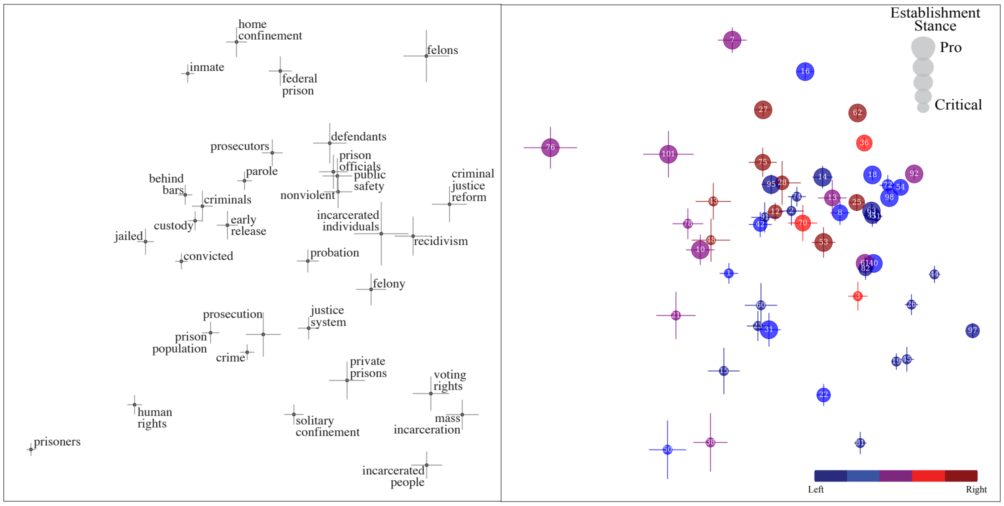

Table S 1. Topic relevance weights for the left-right and establishment topic clusters

Topic Component	Left-Right Weight	Topic Component	Establishment weight
affirmative action 1	0.271	Venezuela 2	0.265
BLM 1	0.245	military spending 2	0.263
Palestine 1	0.235	Palestine 2	0.257
guns 1	0.233	Yemen 2	0.235
tech censorship 1	0.230	police 2	0.228
Venezuela 1	0.226	prisons 2	0.218
abortion 1	0.224	private finance 2	0.216
Russia 1	0.202	nuclear weapons 2	0.215
Yemen 1	0.200	Native Americans 2	0.194
human rights 1	0.200	human rights 2	0.188
climate change 1	0.184	China 2	0.187
sexual harassment 1	0.181	Russia 2	0.180
US immigration 1	0.170	nuclear weapons 1	0.178
church state 1	0.166	Israel 2	0.173
police 1	0.163	espionage 2	0.153
church state 2	0.160	Islam 2	0.152
Judaism 1	0.160	espionage 1	0.148
Israel 1	0.158	public finance 2	0.145
universities 1	0.151	Islam 1	0.136
Judaism 2	0.151	private finance 1	0.135
oil 1	0.128	BLM 2	0.133
tech censorship 2	0.117	oil 2	0.132
climate change 2	0.117	US immigration 2	0.130
universities 2	0.094	guns 2	0.090
public finance 1	0.074	abortion 2	0.088
		China 1	0.083
		affirmative action 2	0.066
		sexual harassment 2	0.054
		Native Americans 1	0.053
		prisons 1	0.049
		military spending 1	0.048

When we performed the generalized singular value decompositions for each topic, we had the freedom to choose both the sign of each plotted component and whether we numbered it 1 or 2. To eliminate these ambiguities and standardize the components, we the automatically flip signs such that all topics k in Cluster 1 have $E_{1k} > 0$ and all topics in Cluster 2 have $E_{2k} > 0$, and numbered the two components as follows. The two components each have a relevance weight as shown in the Table 1: the one with the largest relevance weight is numbered "1" if it is a left-right component and "2" otherwise; the second component gets the opposite number.
